# Clinical diseases with thrombotic risk and their pharmacologycal treatment: How they change the therapeutic attitude in dental treatments

**DOI:** 10.4317/medoral.19561

**Published:** 2013-10-13

**Authors:** Federico Martínez-López, Ricardo Oñate-Sánchez, Juan J. Arrieta-Blanco, Daniel Oñate-Cabrerizo, Maria C. Cabrerizo-Merino

**Affiliations:** 1 Medical Doctor. Odontologist. USBD Medical Center of Fuente Álamo and Mazarrón. Contributor of Docent Unit of Special Patients. University Odontological Clinic. Faculty of Medicine. Murcia; 2 Medical Doctor. Stomatologist. Permanent Professor of University. Docent Unit of Special Patients. University Odontological Clinic. Faculty of Medicine. Murcia; 3 Medical Doctor. Stomatologist. Associate Chief of Odontostomatological Service of the Jiménez Díaz-Capio Foundation. Madrid. Autonomic University of Madrid; 4 Doctor. Contributor of Docent Unit of Special Patients. University Odontological Clinic. Faculty of Medicine. Murcia; 5 Medical Doctor. Stomatologist. USBD Medical Center of Ranero. Associate professor of Docent Unit of Special Patients. University Odontological Clinic. Faculty of Medicine. Murcia

## Abstract

The new antiplatelets and anticoagulant drugs have been recently introduced in the daily medical practices for the control of thromboembolism associated with different diseases. The dental assistance of these patients forces us to know these drugs, understand their action mechanisms and try to decrease the risks that entail ours actions in these patients, making a thorough analysis of the risk of bleeding that is going to be related to our medical intervention, as well as the use of all the control measures of the hemorrhage from our knowledge with these patients, and to be prudent. The communication with the medical specialist that supervises these patients must be maxim, being necessary to make clinic trials for establishing protocols or guides of the handling with these patients during the odontological treatment.

** Key words:**Antiplatelet drugs, anticoagulants drugs, new/classic, thrombotic risk, hemorrhagic risk, dental treatment, caution.

## Introduction

There are a large number of diseases with an increase of the risk of thrombosis. Between them the ones that stand out are: the cardiovascular diseases (ischemic heart disease, peripheral arterial disease, etc.), the neurological diseases (stroke, transient ischemic accident or TIA, etc.), certain arrhythmias like atrial fibrillation (AF), etc., surgeries that order prolonged immobilization, etc. For the prevention and control of these thrombotic risk there has been used the classics antiplatelets drugs (acetylsalicylic acid or ASA, clopidogrel, ticlodipine and dipyridamol) as well as the classics anticoagulants drugs (warfarin, acenocoumarol and heparin). In the last years there have been appearing new anticoagulants and antiplatelets drugs. With these new drugs the doctors have wanted to increase the reaction time, have a better prediction about the effect that is wanted on the patients, reduce the secondary effects and minimize the possible pharmacological interactions of these. In this article we have tried to make a review of the main characteristics of the antiplatelet and anticoagulant drugs with clinic application, as well as establish a guide of the use of the patients with this type of treatment when there are going to be made a dental treatments.

## Bibliographic review antiplatelet drugs

-Classic antiplatelet drugs (1)

The old antiplatelets drugs are a heterogeneous group used for prevent that platelets do their physiological function. The platelets attend principally in primary hemostasis, and in the coagulation phase too. In the platelet activation act different enzymes: the cyclo-oxygenase, the phosphodiesterase and the adenosine diphosphate (ADP). The old antiplatelets act normally on different enzymes and substances, for example the classic inhibitor of the cyglo-oxygenase is the acetylsalicylic acid (ASA), the inhibitor of phosphodiesterase is dipyridamole and the ADP receptors blockers are clopidogrel and ticlopidine. ACETYLSALICYLIC ACID (Adiro®, Bioplak®, Tromalyt®)

Is the non-steroidal anti-inflamatory and antiplatelet most used for the prevention of thromboembolic diseases. ASA produce an irreversible inhibition of the platelet´s cyglo-oxygenase. This enzyme acts in the change from arachidonic acid to thromboxane A2, so we can see a reduction of thromboxane A2´s levels. The action of the thromboxane A2 stimulates the secretion and platelet aggregation. The effect goes on the platelet´s half life, that it is around 7-10 days. The preventive dose is 75-150 mg/day and the therapeutic dose when an immediate antithrombotic effect is demanded is 300 mg/day.

DIPYRIDAMOL (Persantin®)

Produce an inhibition of the phosphodiesterase enzyme. This enzyme catalyzes from cyclic adenosine monophosphate (cAMP) to AMP, so cAMP increases inside the platelet, and cause a block of the platelet activity. It is a reversible effect and goes on 24 hours.

TICLOPIDINE (Ticlodone®, Tiklid®)

It is a platelet ADP receptor blocker. It´s in the thienopyridines group and it was the first. Is a non activated drug that when is activated produce the blocker of the platelet´s ADP receptor. We use this drug in patients with resistance problems to ASA. It has a very important secondary effect: neutropenia that normally is reversible.

CLOPIDOGREL (Plavix®, Iscover®)

It is a thienopyridine with higher antiplatelet action than ASA. It has an irreversible effect during 7-10 days and less secondary effects than ticlopidine so we prefer to use it. It has a problem: is very expensive. We use it mainly in acute myocardial infarction (AMI) alone or with ASA. Sometimes we can see in some patients a variable response including resistance probably for a bad bioavailability (poor absorption, pharmacological interference), alterations in the platelet functions and another factors like smoking, hypercholesterolemia (2).

There are different studies that prove the efficacy of the treatment with the classical antiplatelets alone or dual antiplatelets therapy (3,4). They has a residual risk of ischemic diseases, high risk of bleeding and a variable antiplatelet effect, so it has done that scientist look for a new antiplatelets drugs mainly in the group of receptors p2y12 of the ADP inhibitors and in another groups.

New antiplatelets drugs. Pharmacological properties and therapeutic intructions.

The most important therapeutic condition of the new antiplatelet drugs is to reduce the ischemic events without increase the bleeding risk. The usual indication of these drugs is for the management of thrombotic diseases that we can see in: acute coronary syndromes, ischemic stroke and TIA, peripheral artery disease and percutaneous coronary intervention.

PRASUGREL (CS-747, LY640315) (Efient®)

It is a third generation thienopyridine. Is an antiplatelet drug the most fast and powerful and we can see the effect 30 minutes after it will be administrated (5). It will be introduced in the clinic practice for the thrombosis prevention after of percutaneous coronary intervention (6). The clinic efficacy has been evaluated in the TRITON-TIMI 38 study where we can see the efficacy of prasugrel and ASA compared to clopidogrel and ASA. The results of this study demonstrated than the incidence of cardiovascular death, AMI, and stroke are less, but we observe a major tendency of bleeding, including fatal ending (7), above all in patients older than 75 yeas old, weight less than 60 kilograms and patients with previous stroke or TIA (4) and/or coronary by pass surgery (6), so we must avoid using this drug in this patients (5).

Prasugrel doesn´t have pharmacological interaction with proton pump inhibitors (lansoprazole, omeprazole, etc), and neither with inductors of this proton pump: rifampicin, carbamazepine, ketoconazole, verapamil, diltiazem, ciprofloxacine and clarithromycin. (4,8). It is the first choice in diabetic patients because they show an increase in platelet reactivity and a less response to clopidogrel. By the way, we don´t need to adjust the dose in renal failure (4).

TICAGRELOR (AZD6140) (Brilique®)

It´s a new reversible direct inhibitor of the P2Y12 receptor and is included in the nonthienopyridine group. It has a very fast action (complete platelet inhibition in 2 hours) and the inhibition disappears in 12 hours approximately (3). The efficacy of ticagrelor was evaluated in the PLATO study, with ASA and ticagrelor compared to an ASA and clopidogrel. They observe a less incidence of cardiovascular death, AMI, and stroke. They don´t find a major tendency of bleeding, but they observe after 30 days an increase of bleeding not related with coronary by passes after 30 days (9,10). It has some adverse effects: dyspnea, and increase in serum of uric acid and creatinine (3). Ticagrelor has an oral administration.

CANGRELOR (AR-C69931) (6).

It is an antagonist of the platelet ADP´s receptors. It has a very short answer and produces a reversible platelet inhibition. The way of administration is intravenous, so the effect is immediate. It makes that cangrelor is used when we need antiplatelet effect during perioperative time.

ELINOGREL (PRT060128)

It is a new reversible direct inhibitor of the P2Y12 receptor for the ADP. The way of administration can be oral or intravenous. The effect is immediate if we administrate it intravenously during 12 hours. We can find adverse effects like dyspnea and increase in serum of transaminases (3,6).

OTHERS

Presently a new group of inhibitors of the platelet PAR-1 receptor is being developed (is a thrombin receptor). In this group we can include: terutroban, atopaxar (E5555) y varopaxar (SCH-530348) (3,6).

## Anticoagulant Drugs

-Classics anticoagulant drugs 

HEPARIN

It is a linear mucopolysaccharide, whose action appears when it binds to antithrombin III, accelerating its reaction. The antithrombin III is a potent inhibitor of the thrombin. Other coagulation components can be inactivated like IXa, Xa, XIa, XIIa factors and plasmin. We can measure its effect with the activated partial thromboplastin time (aPTT).

At this moment we use low molecular weight heparin (LMWH) because it has a high half life of 4 hours (normally heparin has a half life of 1-2 hours). It is eliminated completely in 24 hours, making easy the management. It has a parenteral way of administration. We use LMWH in cases we need anticoagulation or thrombosis prevention for a limited time. Due to the disadvantage of the way of administration and the secondary effects appears if it is administrated for a longer period than 6 months (thrombocytopenia, osteoporosis). It has an antidote: protamine sulphate, so we can use heparin for bridge therapy to oral anticoagulants in surgery and prolonged immobilization (11).

WARFARIN (Aldocumar®) Y ACENOCOUMAROL (Sintrom®)

Warfarin and acenocoumarol are oral anticoagulant drugs of the coumarin group. Warfarin is used in Anglo-Saxon countries, and in Spain we normally use acenocoumarol (12).

Its way of action is the same: vitamin K antagonist drugs so they reduce the hepatic synthesis of the factors of vitamin K coagulation: II, VII, IX and X, as well as C and S proteins. Its effect is not immediate but requires several days to get it. As pharmacokinetics qualities: they present a good oral absorption, a high tendency to union to plasmatic proteins, an hepatic and renal metabolism and a half-life between 10 and 24 hours. These characteristics make possible a great number of factors which take part in its absorption and that can modify the potential of action. They also present a great number of drug interactions.

Its anticoagulant effect is measured by the prothrombin time (PT). As there were differences between many laboratories, in 1978 the World Health Organization (WHO) recommended to standardize PT and in 1983 the INR (International Normalized Ratio) was introduced, being the quotient between TP´s patient and the TP standardized by the laboratory or control (13,12). Its normal accepted value for the control of the anticoagulation requested in different clinic entities is around 2 and 3.5, so it´s recommended to be between 2 and 3 in illnesses like the prevention of venous thromboembolism, prevention of systemic embolism in cases of some arrhythmia as AF, etc., and between 2.5 and 3.5 like in the case of heart valve prosthesis, recurrent systemic embolism and recurrent AMI (12).

-New anticoagulant drugs. Pharmacological characteristics and therapeutic indications

In the last years, pharmaceutical industry has developed new anticoagulant drugs, which produce a more selective effect on coagulation components, an easier way of use so a reduction of possible complications. The main indications of new anticoagulant drugs are the prevention of strokes and systemic embolism in cases of some arrhythmia as AF, just like the prevention of venous thromboembolism in patients after elective hip or knee replacement surgery. CHADS2 score is a try to quantify stroke and TIA risk in patients with AF, to value if anticoagulation is required, where C is congestive heart failure, H hypertension, A age > or = 75 years, D diabetes y S stroke or TIA. Each letter punctuate one point, except S that its presence which means 2 points. The scoring range is between 0 and 6. A 2 point score forces the need of anticoagulation. Recently there has been added other risk factors, changing the scale to CHADS2-VASc, where V is vascular diseases, A is the age between 65 and 74 and Sc is female gender. (14). In this group of drugs we can note:

XIMELAGATRAN (15) (Exanta®)

It is the first competitive and direct thrombin inhibitor. Its efficiency and safety were valued in the SPORTIFF III y V study where was compared to warfarin. An identical efficacy was found in the prevention of stroke and TIA in patients with non valvular AF and with some vascular risk factor added. It was also observed that It didn´t have a greatest tendency to bleeding, although It was removed from the market because of its hepatotoxicity.

DABIGATRAN (16) (Pradaxa®)

It´s administrated like dabigatran etexilate (inactive), which is a pro-drug transformed to dabigatran (active) by the hydrolysis of plasmatic and hepatic esterases. It is a competitive and reversible thrombin inhibitor, inhibiting the reaction from fibrinogen to fibrin, so inhibits the thrombus formation. So it also inhibits free thrombin, thrombin linked to fibrin and antiplatelet effect induced for thrombin. In Spain we use it for: primary prevention of venous thromboembolism in patients after elective hip or knee replacement surgery, , stroke prevention and systemic embolism prevention in non valvular AF in patients with one or more of these factors: stroke, TIA or previous systemic embolism, ventricular ejection fraction < 40%, symptomatic heart failure > or = Class II of the New York Heart Association (NYHA) scale, age > or = 75 years and age > or = 65 years related to one of the next diseases: diabetes, coronary disease or hypertension.

Efficiency and security have been evaluated in RE-LY study, where is compared dabigatran etexilate from warfarin in patients with AF with moderate or high risk of stroke, TIA or systemic embolism, with two doses: 110 mg/twice a day and y 150 mg/twice a day. They have demonstrated a similar efficiency to warfarin without higher bleeding tendency with doses of 110 mg/twice a day. With doses of 150 mg/twice a day, they have demonstrated a higher efficiency in reduction of ischemic and hemorrhagic stroke, vascular death, intracranial hemorrhagic and total bleeding compared to warfarin, although the AMI indexes were higher. The secondary effect more frequent was bleeding in a 16,5 % of the patients. Other adverse effects were: epistaxis, anemia, gastrointestinal haemorrhage, abdominal pain, diarrhea, dyspepsia and nausea (17).

It has a renal elimination above all, so the renal function will be controlled. In case of renal failure increases the incidence of bleeding. Other factors that increase hemorrhagic risk are: age >75 years, low bodyweight, pharmacological interactions, etc. Dabigatran is contraindicated in mechanical heart valve prosthesis, severe renal failure with creatinine clearance < 30 ml/min. We need to be careful in patients with moderate renal failure (createnine clearance between 30-50 ml/min).

If we administrate dabigatran with glycoprotein-P inhibitors (amiodarone, verapamil, quinidine, ketoconazole and clarithromycin) can increase the dabigatran’s effect, and if we administrate it with glycoprotein-P inducers (rifampicin, St. John, s herb, carbamazepine, phenytoin) the effect decreases.

Dabigatran changes coagulation tests like aPTT, PT, thrombin time (TT) and ecarin time (in experimental phase). In case of emergency we can use the TT to value the dabigatran’s effect (15).

New anticoagulants lack specific antidote, so in emergency situations that need reduction or anticoagulation´s cessation (important haemorrhages, etc.), makes difficult its use. In the case of dabigatran it is been proposed the administration of active carbon for reducing the absorption of the drug, and the use of hemodialysis for increasing its elimination (15,18). It’s been also suggested the administration of blood transfusion or fresh plasma (19).

RIVAROXABAN (Xarelto®)

It´s a competitive, reversible and direct inhibitor from coagulation factor Xa (17).

 Its therapeutic indications in the United States and in Europe include: prevention of venous thromboembolism in patients after elective hip or knee replacement surgery, prevention of stroke , TIA or systemic embolism, non valvular AF with one or more of the following factors: congestive heart failure, hypertension, age > or = 75 years, diabetes, stroke or TIA previous. Nowadays it´s been investigated its use in the treatment of lung thromboembolism and the prevention of the recurrent episodes (17).

Its efficiency and safety have been valued in the ROCKET AF study, where it´s compared to warfarin in patients with non valvu-lar AF, moderate or high risk of stroke or systemic embolism.

It lacks a valid monitoring method for testing its efficacy, altering the aPTT and even more the PT, so this last one is used for quantifying its effect (15).

APIXABAN (Eliquis®)

It action consist, as the rivaroxaban, in a selective inhibition of the coagulation factor Xa. It´s been investigated its use in the treatment of deep venous thrombosis, acute pulmonary thromboembolism and prevention of recurrent episodes. (17). It´s been also valued its use in unstable angina for preventing cardiac ischemic episodes.

Its efficacy has been proved in two recent studies: AVERROES and ARISTOTLE. These studies conclude that apixaban presents a higher efficiency in the prevention of stroke and systemic embolisms than ASA and warfarin respectively, not objectifying a greater bleeding incidence and less mortality.

EDOXABAN (Lixiana®)

It is a direct and specific inhibitor of factor Xa. It´s been investigated its use in deep venous thrombosis, pulmonary thromboembolism, and in the prevention of embolic episodes in patients with AF. It´s widely eliminated by urinary tract so its dose must be settled in case of renal failure (20).

BETRIXABAN

It is a direct inhibitor of factor Xa. It´s been investigated its use in the prevention of embolic episodes in patients with AF. Its main elimination is the biliary tract, only eliminating 5% through the urine so it could be indicated for patients with renal failure (20).

Every direct inhibitors of coagulation factor Xa, previously enumerated, have an oral way of administration (17).

OTAMIXABAN

It´s a potent and selective inhibitor factor Xa with an intravenous way of administration (17).

## Odontostomatologial treatments in patients with classic antiplatelets and anticoagulantspautas 

-Dental management in patients with classic antiplatelets.

The attention guidelines in the dental cabinet of patients with classic or new antiplatelets, don´t differ at all considering that there aren´t any studies with the new drugs. We have to compare the measures of the old ones to the new generation. In the bibliographic revision we haven´t found randomized essay not even cohort studies where the dental management of patients under a treatment with new antiplatelets is valued and limited studies where the classic antiplatelet are valued in the dental practice (21). We agree with the material published in the Guidelines of the American College of Chest Physicians about perioperative management of antithrombotic therapy (22).

-Dental management in patients with classic anticoagulants.

Throughout the history of the use of anticoagulant drugs in the medical practice, it has been seen an evolution in protocols of dental management of these patients. At first there were protocols that recommended replacing the anticoagulant drug (warfarin or acenocoumarol) for low molecular weight heparin (LMWH) with different possibilities.

In these patients we must considerate the risk of thrombosis if we suppress the anticoagulant therapy, facing the risk of bleeding if we don´t do it. In the review article of Pototski et al. (1), marks that the thrombosis tendency in the different revised studies is between the 0.02% and the 1% for minor methods like dental extraction meanwhile the risk of bleeding is between the 0% and the 3.5% according to the studies. The problem is that the possibility of fatal ending is more probable in the case of thrombosis appearance, than in the case of haemorrhage. Because of that, nowadays the studies recommend that in case of simple dental extraction of one or two teeth (contiguous or adjacents), not removing the anticoagulant drug (1,12,13,23-25). We must control the drug´s anticoagulant action with the INR, whose value has to be done at least 72 previous hours to the dental extractions, although it´s better if it´s done 24 hours before. The INR ideal value for doing the dental extractions is established in different studies between 2 and 4, although it´s widely accepted that the optimal value is in 2, 5 because this value minimises the risk of bleeding and thrombosis (1).

If we think that the dental extraction will be complicated, flap and osteotomy is necessary or patients with multiple pathologies we can bridge anticoagulants with LMWH 2 or 3 days before dental surgery. We can do the same in patients with high thrombosis risk (24).

With these patients we must use local hemostatic actions like (1,12,23,25):

- Previously we have to remove irritation and inflammation of oral tissues, because bleeding tendency is higher. We can use oral hygiene tips, tartrectomy, chrohexidine rinses, etc.

- We must do the dental extraction carefully trying to eliminate the less amount of bone tissue.

- Curettage of the alveolus to eliminate granulation tissue.

- Irrigation of the alveolus with antifibrinolytics like tranexamic acid.

- Use hemostatic sponges with fibrinogenous elements.

- Suture the wound with resorbable sutures preferably. If we use non resorbable sutures we have to remove in 4-7 days.

- Do compression with gauzes soaked with antifibrinolytics like tranexamic acid.

- Bleeding control of the dentist. The patients must stay in the clinic alter the dental extraction at least 45-60 minutes.

- Mouth rinses with tranexamic acid.

- Patient’s control in days after surgery.

-Give writing post-extraction instructions (most of them were made by Scully y Cawson (1) including: control clot, not rinsing the mouth for 24 hours, not suck or manipulate the clot with the tongue, not to take hot drinks or hard food, etc. In case of bleeding the patients has to put a clean gauze soaked with antifibrinolytics like tranexamic acid and make pressure; if after 20-30 minutes the bleeding doesn’t stop, the patient has to contact with the dentist. For this reason it is recommended to do the dental extractions in the morning and in the beginning of the week.

## Variations of classic odontostomatologial treatment guidelines in patients with new antiplatelets and anticoagulants

-Dental management in patients with new antiplatelets.

In the dental management of the patients with antiplatelets treatment we have to value two factors: the bleeding risk of dental treatment and the thrombotic risk of the disease that has needed antiplatelet treatment. The risk of bleeding conditioned that we have to use local hemostatic actions or not. The thrombotic risk conditioned the possibility of modifying antiplatelet treatment.

The principal action with bleeding risk is the dental extraction. Normally a simple dental extraction has a low bleeding risk while a multiple dental extractions have a high risk of bleeding (24). In the case of mucogingival surgery is low risk of bleeding if it isn’t very extensive. Sometimes is difficult to predict if the dental extraction will be simple or complicate. If there is an inflammatory process the risk of bleeding will be high (24).

The thrombotic risk will be established for the medical specialist of the patient because there are lots of factors that can condition it like: underlying disease, age, time from acute episodes, renal function, good medical control, etc. So the decision of removing or not the antiplatelets treatment will be done by the medical specialist and not by the dentist or by the patient.

In the review article of Hall R. y Mazer CD (6), about the perioperative use of patients under an antiplatelet treatment, indicate that in the case of existing during a surgical intervention, a risk of low bleeding and the risk of thrombosis (in this case: stent thrombosis) being high or moderate, they recommend not interrupting the antiplatelets treatment, but if the risk of thrombosis is low they recommend suppressing some of the antiplatelet treatment in the case of being dual and recovering it as soon as possible. In the guide of the European Society of Gastrointestinal Endoscopy for the management of patients with antiplatelet agents that need an endoscopy (26), made by Bosutière C, et al. They note that in methods with low risk of bleeding and risk of thrombosis like any other, it´s recommended continue with the antiplatelet treatment. In case of methods with high risk of bleeding, they note removing the pharmacological antiplatelet treatment or replacing it, asking first the medical specialist. The reflected information in both studies could be comparable to dental management of patients when we are going to perform a simple dental extraction, considering it a surgical act of low risk of bleeding (24).

There are numerous studies and publications where not suppressing the antiplatelet treatment is recommended in case of a surgical act with low risk of bleeding and especially in the case of a simple dental extraction (27,28,29). It´s also of importance that most of the published studies have been produced at patients who were having ASA, clopidogrel or both, because these two are the most used antiplatelet drugs nowadays. (27-29).

We can establish as a possible practice guide of dental management in patients under an antiplatelet treatment.

- Establish the risk of bleeding. Simple dental extraction as low risk and multiple extractions as high risk (24). It´s been deter-mined in some studies the relation between the number of extractions and the bleeding tendency: in the study of Cardo-na-Tortajada F, et al. (28) established a connection statistically significant between the bleeding and the number of extracted teeth, remarking that it´s not recommended to perform more than 3 dental extractions in the same surgical act and that these must be of adjacent teeth.

- Establish the thrombotic risk of the patient by the medical specialist who controls the patient.

- Application of local measures for the control of the haemorrhage indicated before.

-Dental management in patients with new anticoagulants.

The new anticoagulant drugs, as we have mentioned before, present advantages and disadvantages from classic vitamin K antagonist. Zapata Wainberg G et al. (15), in the advantages they find its few pharmacological and dietetic interactions, not requiring the monitoring of routine, its quick start of action, having less or similar bleeding rates, etc. In the disadvantages they also note not having specific antidotes in case of emergency situations for reversing the anticoagulant effect, a high price, some of them must be administrated two times a day and the lack of valid methods of monitoring for quantifying its effect.

There have been published many articles about dental management with patients under treatment with classic anticoagulants (1,12,30). However, there are only a few studies about dental management in patients under treatment with the new anticoagulant drugs. In the last 12 months have appeared in medical literature some articles trying to establish a guideline for these patients, when we have to do them a dental treatment, although they also indicate the need of making clinic studies that support the protocols.

We agree with the article of Spyropoulos AC. and Douketis JD. (24) about the risk of bleeding, considering that simple dental extraction and minor surgery have a low risk of bleeding, however the multiple dental extractions have a high risk of bleeding, for this we recommend the dental extraction no more than 3 teeth in the same surgical act and they will be contiguous or adjacents . It is better if we do simple dental extraction (1,28). Because of all, considering that usually our acts are in low risk bleeding, and that there are numerous studies that recommend not removing anticoagulant anti vitamin K (1,12,30), we could think that with the new anticoagulant drugs (dabigatran, rivaroxaban, etc.) it would not be necessary removing its during the dental treatment (31,32), although, as we have indicated previously, there are not any studies that support this particular way of acting, and we must be prudent.

We classify the thromboembolisn risk in patients with the new anticoagulants (remember that the main indication is non valvular AF) in 3 groups (24): the risk is high when we have a CHADS2 score of 5 or 6, stroke or recent TIA (< 3 months) or history of rheumatic valve disease. A moderate risk if CHADS2 score is 3 or 4. Finally the risk is low if CHADS2 score is between 0 and 2 and there isn’t previous stroke or TIA.

Combining both factors we could say that in cases of high thromboembolism risk and low bleeding risk we shouldn’t remove new anticoagulant treatment (31,32). In case of low thromboembolism risk and high bleeding risk, the medical specialist who controls the patient should modify the new anticoagulant treatment. An advantage of new anticoagulants (dabigatran, rivaroxaban and apixaban) is that they have a reduced half life in patients with normal renal function, and in case of being necessary its removal, it will be done 24 hours before the surgical treatment. If renal function is wrong the removal will be done between 4 days and 24 hours in the case of dabigatran, and between 2 days and 24 hours in the case of rivaroxaban, before surgery (19). Its reintroduction must be done as soon as possible if there isn’t bleeding, normally recommending to do it 24 hours after surgical act. If there is a postoperative bleeding risk the reintroduction will be done after 2-3 days from surgery (24) with normal dose. Other authors recommend the reintroduction as soon as possible with minimun bleeding surgery: 4-6 hours after the act, with half normal dose and increase it gradually (19). By the way it will be controlled for the medical specialist. With these patients we must use local hemostatic actions for minimizing bleeding risk.

As some authors noted (33), in the dental treatment of anticoagulated patients we have to value different factors: kind of surgery, analytical values, medical specialist recommendations, oral and medical conditions of the patients. All of them must be considered before dental treatment, to minimize potencial risks and to get success in our treatments with them.
